# Response of Human Red Blood Cells to Acute and Chronic Oxidant Challenge as Observed Through the Glutathione and Glutathionyl-Hemoglobin Redox Pairs In Vitro and In Vivo

**DOI:** 10.3390/molecules31050811

**Published:** 2026-02-28

**Authors:** Federico Maria Rubino

**Affiliations:** Department of Health Sciences, University of Milano, v. A. di Rudinì 8, I 20142 Milano, Italy; federico.rubino@unimi.it

**Keywords:** 1,3-butadiene, busulfan, chronic oxidative stress, glutathione-dehydroalanine, glutaredoxin, electrochemical potential, glutathione-S-transferase, phase space, *t*-butyl-hydroperoxide, tobacco smoking, thioredoxin, thioredoxin-reductase, transient oxidative stress

## Abstract

Glutathionyl-hemoglobin (HbSSG) reversibly forms under oxidative stress in erythrocytes, where it constitutes the main redox buffer, in a dynamic equilibrium with the thiol (GSH) and disulfide (GSSG) forms of glutathione, that quickly revert to the reduced thiols when oxidative pressure is relieved. Under acute challenge, the “oxidized” GSH pool distributes between GSSG and HbSSG. Recalculation with electrochemical metrics based on redox potentials of the GSSG/GSH and HbSSG/HbSH pairs, plotted in their phase space, improves the understanding of the competing reduction processes. The first process is reduction of the GSSG pool, while, later, HbSSG reduction occurs as a two-step process. HbSSG accumulation in chronic oxidative stress follows an impairment of these steps. In 30 strong smokers, homogeneous levels of HbSSG are in the range of 2.4–11.7% (E_h_ −120–−95 mV), but the E_h_ of the GSSG/GSH redox pair is wider (−160–−240 mV), suggesting that HbSSG accumulation does not depend on GSH availability but on enzyme activity impaired by exogenous and endogenous electrophiles. As hinted by HbSSG measurements, one such species is the dehydro-alanine analog of GSH, produced both from butadiene in exposed petrochemical workers and from the drug busulfan in a treated patient. Inactivation of the low-copy recycling enzymes can thus explain the increase of HbSSG.

## 1. Introduction

Glutathionyl-hemogobin (HbSSG) is an increasingly promising molecular biomarker of systemic oxidative stress, due to its specific and direct measurement in red blood cells (RBCs) with different techniques and to the stoichiometric relationship with other redox nodes of the erythrocyte, in particular the glutathione-glutathione disulfide and cysteine-cystine nodes [1]. Recent advances include thorough molecular and biophysical characterization [2,3,4,5], increasingly improved analytical methods [6,7,8,9], and more articulated hints at its generation mechanisms under different conditions in vitro [10,11,12] and in vivo [13]. This increased knowledge encourages widened applications in the characterization of systemic oxidative stress in an array of clinical and life conditions.

Several intriguing phenomena of oxidative stress that occur in vivo and in oxidant challenge experiments in red blood cells can be re-interpreted when more than one component of the cellular redox circuitry is taken into consideration.

Synoptic consideration of the two main inter-playing redox buffers of the RBC, viz. those of the glutathione disulfide/glutathione and glutathionyl-hemoglobin/hemoglobin pairs, may shed considerable insight on the oxidant challenge mechanism(s) within the erythrocyte. The interpretation of redox homeostasis in cells follows two “extreme” points of view. One considers the redox reactions in plasma and in the cytoplasm of cells and organelles as described by the respective electrochemical potentials, which can be in turn calculated with the Nernst equation from the standard redox potential of the participating species and considering their respective actual concentrations (activities) [14]. A strong caveat to this excessively simplified, “thermodynamic” interpretation warns that “uncatalyzed” thiolate exchange under physiological conditions is exceedingly slow and unable to explain the fast response of living compartments to the changing conditions of oxidative challenge. Therefore, the relative levels of the soluble thiols in the thiol(ate) and disulfide forms are regulated by the enzymic activities of several interplaying proteins, rather than by sheer thiolate exchange reactions [15].

This work exploits the availability of results from sets of samples, where the levels of glutathione, glutathione disulfide and glutathionyl-hemoglobin were simultaneously measured in oxidatively challenged red blood cells both ex vivo in vitro and in vivo and from other published work. Re-evaluating this body of work aims for a better understanding of the generation processes that lead to HbSSG formation and degradation, in the view of positioning HbSSG within the panel of available biomarkers of oxidative stress.

Re-examining the available results using the calculated redox potential (E_h_’) as a biomarker, rather than individual analytical concentrations, can contribute to addressing some as yet still open questions on the biochemical behavior of RBCs subject to acute and chronic oxidative stress. Further insight can be achieved by re-examining HbSSG levels in subjects exposed to specific chemical toxins that are able to selectively impair specific biochemical processes that employ thiol-containing enzymes. In particular, the forwarded hypothesis is that, while continuous production of HbSSG can derive from multiple mechanisms that involve acute or chronic exposure to direct or indirect oxidants, its measured levels are determined by the efficiency of the antioxidant network of the RBC to reduce the mixed disulfide, especially by means of its glutathione-recycling enzyme network.

One outcome of this study is the possibility to explain the differences in the response of RBCs to different real-life conditions that involve acute or chronic exposure to different oxidative stressors, which entail reversible modification in the levels of HbSSG, considered as a biomarker of systemic oxidative stress and of RBC resilience to environmental, lifestyle and occupational stressors.

## 2. Results

The results of separate and independent experiments are described and re-analyzed sequentially.

The first study concerns the results of an experiment of single-bout oxidative stress [16] that have been previously re-analyzed in an earlier publication [17], and a second-tier advanced re-analysis is reported here to extract further information aimed at detailing the recovery processes.

The second study re-evaluates some partially reported data of an observational–interventional study in a heavy smokers’ cohort [18,19], with reference to a specific analysis of the thiolome pool, for which the levels of HbSSG were already published [20].

The third and fourth elaborations concern an unreported case of treatment of in vivo treatment with an alkylating agent, compared to the exposure of a human population to a known environmental and occupational carcinogen with similar chemical and toxicological behavior [21].

### 2.1. Ex Vivo, In Vitro Acute Oxidant Challenge of Human Red Blood Cells

A now classic experiment of oxidative challenge of ex vivo, in vitro red blood cells involved burst treatment with the single-strike radical generator *t*-butyl-hydroperoxide, followed by measurement of the levels of glutathione, glutathione disulfide and glutathionyl-hemoglobin [16]. Numerical re-analysis of the reported results under the “thermodynamic” model afforded a value of −121 mV for the standard electrochemical potential of the β-93-cysteine group of human hemoglobin (E_0′ HbSSG/HbSH_) [17], the protein functional group that, upon oxidative challenge, combines with glutathione to generate glutathionyl-hemoglobin [12].

This calculated electrochemical constant was thus used to convert the measured intra-erythrocyte concentrations of glutathione, glutathione disulfide and glutathionyl-hemoglobin into the respective redox potentials for the two pairs, glutathione disulfide/glutathione and of the glutathionyl-hemoglobin/glutathione, during the pre-challenge, challenge and recovery process.

**Figure 1:**
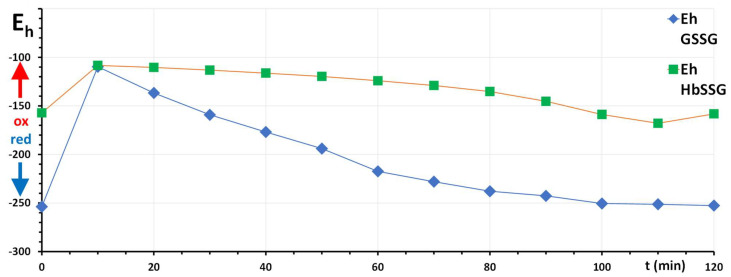
Values of the E_h_ potentials of the redox pairs of GSSG/GSH (blue) and HbSSG/HbSH (green); at different times during the RBC oxidative challenge (red arrow) and recovery phases (blue arrow) of the experiment.

The graph of Figure 1 shows the recovery process using the E_h_ metrics for the two redox pairs, GSSG/GSH and HbSSG/HbSH, where both redox potentials return to the pre-challenge condition over the two-hour recovery time of the experiment. Using the E_h_ metrics, rather than individual concentrations, in this time plot lowers the number of variables from four (the separate concentrations of GSSG, GSH, HbSSG and HbSH plotted against time) to two and, furthermore, highlights the pairwise relationship of the oxidized/reduced pair of each.

**Figure 2:**
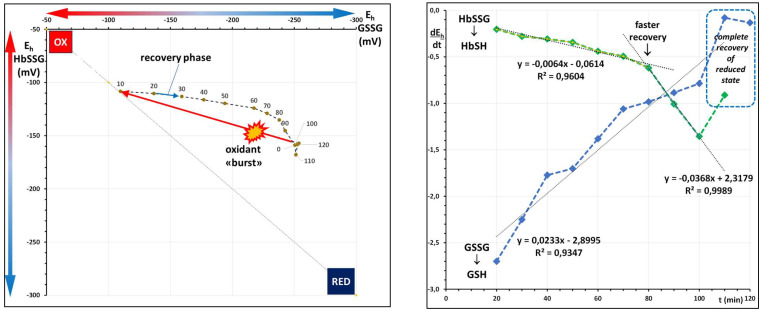
Panel (**left**): Values of the E_h_ potentials of the redox couples of GSSG/GSH (horizontal axis) and HbSSG/HbSH (vertical axis) at different times during the RBC oxidative challenge and recovery phase of the experiment. Each value is labeled with the respective experiment time (closed circles, time in min, numbers close to the closed circles, time flow identified by the linking line). Panel (**right**): Plot of the algebraic first derivative of the recovery plot (dE_h_/dt) of GSH (blue line and symbols) and of hemoglobin (green line and symbols) corresponding to the dotted line of panel (**left**).

The redox potential metrics can be exploited further, in a more insightful way, to investigate the relationship between the two redox pairs during the recovery phase. A scatter plot of the two redox potentials over the experiment time is reported in Figure 2 (left panel, data replotted, analogous to Figure 5 of [17]) and shows the time course of the recovery from the one-time oxidative insult in the phase space of the redox potentials. The reference situation and this powerful data plot have already been used to localize the redox status of other RBCs, including those derived from a subject with an S-variant hemoglobin [22].

The time profile of the two intracellular redox potentials shown in the phase space (left plot) of Figure 2 can be exploited to show the relative timing of the two concurrent redox processes that restore the glutathione pool of the RBC in the pre-challenge condition. Recovery of the glutathione disulfide pool occurs earlier in the time plot (“drop” of E_h (GSSG/GSH)_ from −110 mV to −210 mV between 10 and 60 min), with a negligible change in the E_h (HbSSG/HbSH)_, this process being faster from 60 min to 120 min (complete return to starting conditions).

The occurrence of a two-phase recovery phenomenon can be better envisaged with the calculation of the algebraic first-derivatives of the time changes of E_h_ for the two redox couples, as plotted in Figure 2 (right panel), where it should be recalled that “zero” derivative (at the top) corresponds to “no-change”, i.e., to the essential recovery of the pre-challenge condition, while recovery occurs with a displacement of each E_h_ potential to more negative values, thus with negative values of the derivative, the higher in absolute value meaning a “steeper” decrease, or a faster reduction.

As apparent in the right-side plot, the recovery from oxidative stress of the glutathione disulfide pool is faster at the beginning of the recovery phase and progressively slows, with an almost linear decay, until the end of the experiment. The reduction of the HbSSG/HbSH pool follows a bi-modal trend, with only slow reduction in the early phase (a shallower slope, −0.0064 mV/min, of the first derivative line) and a later much faster reduction (a six-fold steeper slope, −0.0368 mV/min), until the complete recovery. The slope change occurs at approximately 80 min, when most of the glutathione disulfide has been reverted to the free-thiol form. The bi-modal reduction of glutathionyl-hemoglobin is different from the essentially mono-phasic recovery displayed by the GSSG/GSH redox pair (intermediate slope) and may hint that sufficient “reduced” glutathione should be available intracellularly, before the reduction of HbSSG can proceed at sufficient velocity.

### 2.2. In Vivo Chronic Exposure to Active, Strong Tobacco Smoking

In this research, a 30-subject pilot cohort of strong smokers, refractory to smoke-quitting strategies, were encouraged to enroll in a risk-mitigation program that involved daily assumption of vegetable concentrates as nutritional antioxidant supplements [18,19]. In addition to other health checks, the study measured some biomarkers of oxidative stress that include the measurement of the soluble thiolome and of glutathionyl-hemoglobin at enrollment and after a 90-day supplementation [20].

**Figure 3:**
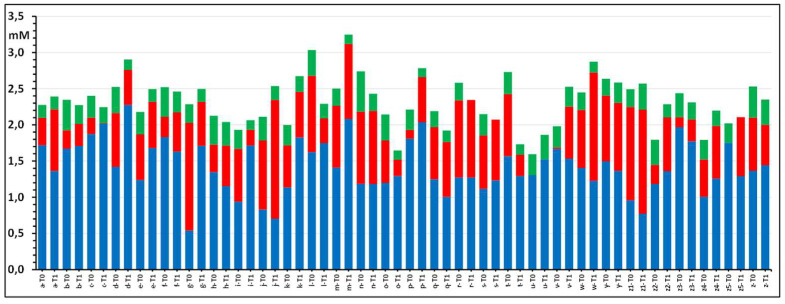
Concentrations of glutathione (GSH; blue), glutathione disulfide (GSSG; red) and glutathionyl-hemoglobin (HbSSG; green) in the red blood cells of 26 strong smokers enrolled in a risk-reduction protocol. Consecutive bars display results of measurement at enrollment (T0) and after the 90-day treatment (T1).

The histograms of Figure 3 show the measured levels of the individual thiolome metabolites in the investigated subjects, at recruitment (T0) and at follow-up (T1), as the cumulative sum.

While glutathione and glutathione disulfides are obtained analytically as intra-erythrocyte concentrations, raw fractional glutathionyl hemoglobin levels need to be converted into concentrations, as reported in [17]. As judged by the sum of the glutathione concentration in the three forms (glutathione, glutathione disulfide and glutathionyl-hemoglobin), the median total glutathione is 2.33 mM (1.59–3.25 mM). This inter-individual variability is, as expected, within the limits of the genetically controlled inter-individual variability reported in the literature [21]. The T1–T0 difference highlights an increase of total glutathione levels in 16/30 subjects (3–34%; median 17%), while 14/30 show a reduction (−2–−37%; median −7%).

**Figure 4:**
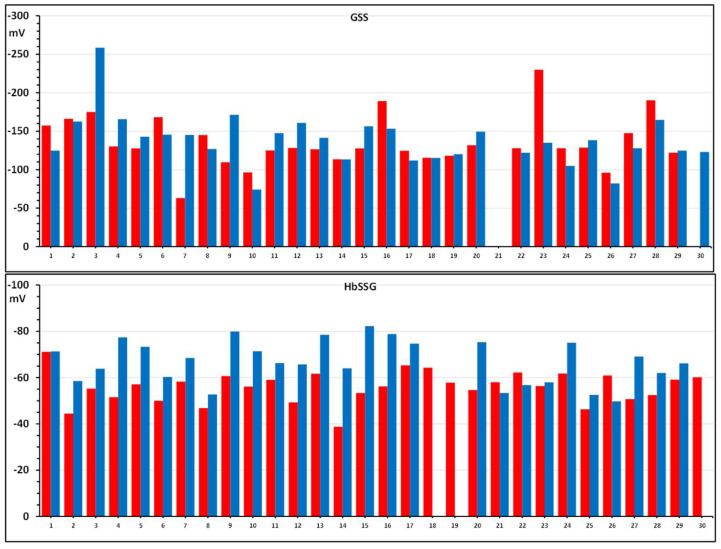
Values of the E_h_ potentials of the redox couples of GSSG/GSH (**upper graph**) and HbSSG/HbSH (**lower graph**) in the red blood cells of 30 strong smokers enrolled in a risk-reduction protocol. Consecutive bars display results of measurement at enrollment (T0; red) and after the 90-day treatment (T1; blue).

The intra-erythrocyte concentrations of glutathione, glutathione disulfide and glutathionyl-hemoglobin are then used to calculate the respective values of redox potential, as plotted in the bar graphs of Figure 4. Only 26/30 subjects have all four measurements necessary for the complete elaboration of the E_h_′ calculation.

**Figure 5:**
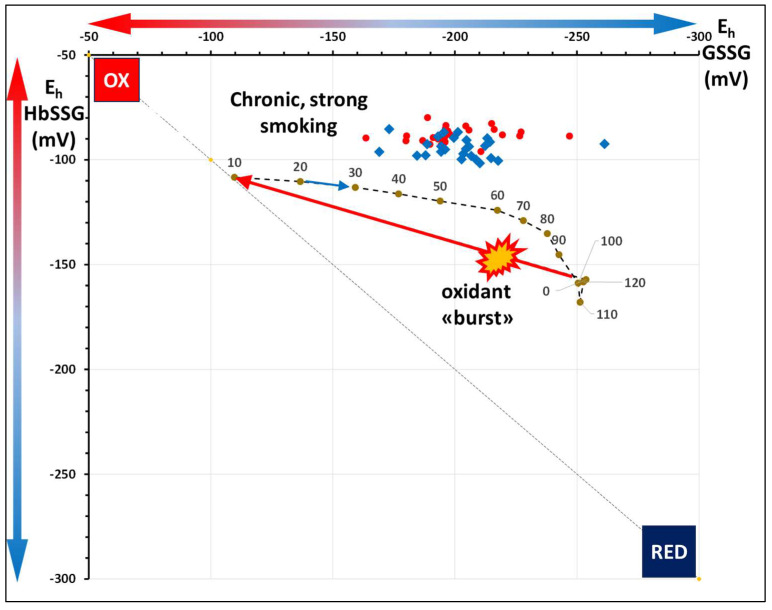
Values of the E_h_ potentials of the redox couples of glutathione disulfide/glutathione (GSSG/GSH); horizontal axis) and glutathionyl-hemoglobin/hemoglobin (HbSSG/HbSH; vertical axis) of 26 strong smokers at enrollment (T0; red) and after the 90-day treatment (T1; blue) with concentrated antioxidants. Shown as reference (closed green dots and numbers) are the E_h_ values of the two redox pairs in the oxidative burst-recovery experiment of Figure 2 (left).

Next, the calculated results are plotted in Figure 5, again with the reference recovery curve (Figure 1) for comparison. This data presentation allows us to consider the condition of chronic oxidative stress of smokers in comparison to that of a transient burst of oxidative stress in a reference healthy subject.

As apparent from the position of the redox pairs of the smokers’ cohort with respect to the single-burst oxidant challenge experiment, the interval of glutathione E_h_ covered by the smokers (−160 mV to −260 mV) spans most of the E_h_ values during the recovery phase of the experiment. On the contrary, the E_h_ of the HbSSG/Hb pair is much higher (more oxidized status), as mirrored by higher HbSSG levels (2.4–11.7% vs. a max of 1.3% just after the ox burst) and by values of E_h_ between −80 and −100 mV. The imbalance of the two E_h_ suggests that recovery of the HbSSG/HbSH compartment, rather than that of GSSG/GSH is more impaired in the strong smokers. This finding also suggests that the strong smokers experience a higher level of chronic oxidative stress in their RBCs than experimentally challenged erythrocytes from a “healthier” non-descript subject.

**Figure 6:**
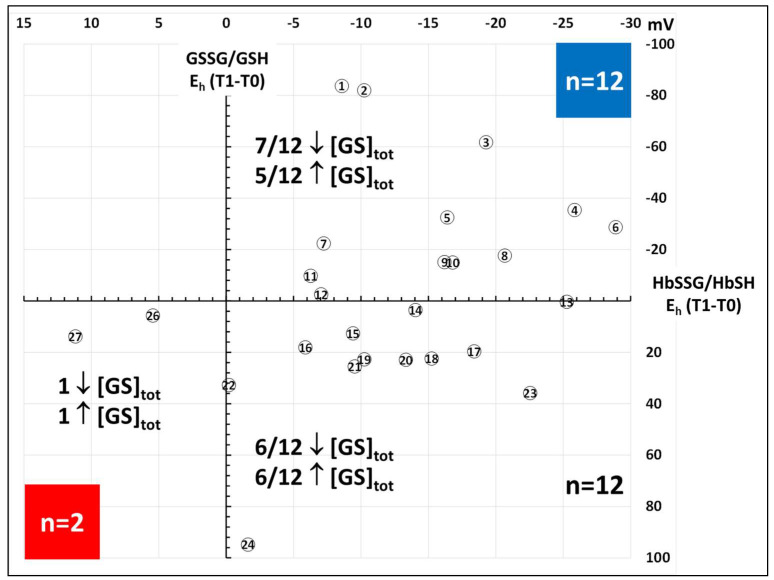
Values of the (T1-T0) difference of E_h_ potentials (mV) of HbSSG/HbSH (horizontal axis) and GSSG/GSH (vertical axis) in the red blood cells of 26 strong smokers at enrollment (T0) and after the 90-day treatment (T1).

To improve data interpretation, the scatterplot of Figure 6 shows the response of each of the 26 subjects between T1 and T2 as expressed by the respective differences of E_h_ for the redox pairs of hemoglobin (horizontal axis) and glutathione (vertical axis), where a negative displacement (to the right for hemoglobin and upwards for glutathione) means an overall reduction process taking place in the redox pairs of hemoglobin and glutathione, respectively.

As measured through the HbSSG/HbSH redox pair, 22/26 subjects improved their oxidative stress status, measured by a decrease of the E_h_ (−0.2 mV–−28.9 mV; median −13.7 mV). Of these, 12 also showed a displacement of their E_h_ GSSG/GSH towards more negative values (−25.6 mV–−83.7 mV; median −2.6 mV; quadrant with the blue square), while 12 moved towards more negative values (+0.3 mV–+94.8 mV; median +22.6 mV). Only two subjects showed a pro-oxidant increase of both E_h_: +5.4 mV; +11.2 mV for HbSSG/HbSH and +5.7 mV; +13.8 mV for GSSG/GSH, respectively (quadrant with the red square). As highlighted in the insert of Figure 6, there is no relationship between the increase/decrease of the total glutathione pool in the subjects (paired T0, T1 consecutive bars in the histogram of Figure 3) and their respective distribution in the quadrants of Figure 6.

It may be worth noting that, after 90 days, most RBCs of the blood pool that yielded the T0 measurement were naturally cleared from the circulation, since the normal lifespan of RBCs is 120 days. Therefore, the “new” RBCs that constitute most of the pool sampled at T1 had less time to accumulate smoke-related chemical damage to the mostly irreplaceable biological structures, especially to the antioxidant enzyme pool.

### 2.3. Exposure to Specific Chemical Agents Can Explain Accumulation of HbSSG

The specific mechanism whereby glutathionyl-hemoglobin can accumulate in RBCs without being promptly reduced is still a matter of debate. Some of the literature and new data may help to shed light on this question.

One peculiar group of electrophilic chemical compounds shares important common features in the reactivity with glutathione. The pharmaceutical drug busulfan (1,4-butanediol-dimethanesulfonate) is a cytotoxic bis-alkylating drug used in cancer therapy to eliminate the diseased hematopoietic cells of patients with leukemia or lymphoma (myelo-ablation) prior to allogenic Bone Marrow Transplantation therapy [22,23]. 1,3-butadiene is the C-4 conjugated olefine produced in the petrochemical industry as a monomer of several polymers, including synthetic elastomers. Its biotransformation products include bis-epoxide (BDE), which is considered one of the most potent chemical carcinogens [24].

#### 2.3.1. Patient Treatment with Busulfan

**Figure 7:**
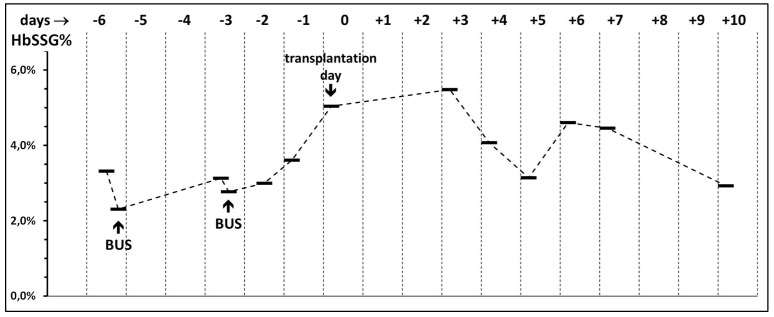
Time course of the levels of HbSSG in a patient treated with injectable busulfan prior to Bone Marrow Transplantation and during the 17-day hospital stay. Events and corresponding days (day 0 being that of the transplantation infusion) are described in Table 1.

This measurement of HbSSG refers to one Bone Marrow Transplantation patient, where blood samples were routinely taken for clinical follow-up and further used as a pre-pilot feasibility test for future study. Typical doses of injectable busulfan are 0.8–1.2 mg/kg_bw_, which correspond to blood levels of 0.8 mcg/L (peak, 1 h) and 0.4 mcg/L (6 h post-infusion) [25,26]. Table 1 describes the treatment schedule of the patient and the measured HbSSG levels, the time course of which is shown in Figure 7.

As observed, the levels of HbSSG change over the investigated period, and, in particular, they double after the second Busulfan infusion and during the post-Busulfan transplantation period, and they start returning to pre-Busulfan levels at day +5, when the patient’s original RBCs are gradually cleared from the circulation and substituted by those generated by the expanding infused clones of healthy hematopoietic blood cell precursors that do not experience hematotoxic effects from the now cleared busulfan. At patient discharge (day +10), the level of HbSSG has returned to that of pre-admission.

#### 2.3.2. Chemical Plant Workers’ Exposure to Butadiene

An important group of independent data derives from the only study in the literature where HbSSG has been measured within a combined workplace and biological monitoring campaign at a petrochemical plant where butadiene is produced [27].

As standard workplace monitoring protocols dictate, butadiene airborne concentration was monitored for the following two groups of plant workers: petrochemical plant employees (n = 42) and plant office workers (n = 43), with an “un-exposed” group of rural population (“Foresters”) as reference (n = 24). All subjects also supplied blood samples where biological markers of exposure and response, including glutathione-S-transferase (GST P1−1 and GST T1-1) activities and glutathionyl-hemoglobin levels, were measured. This re-analysis retrieves and re-plots the data from the publication (Table 2 and Figure 1 of [27]).

**Figure 8:**
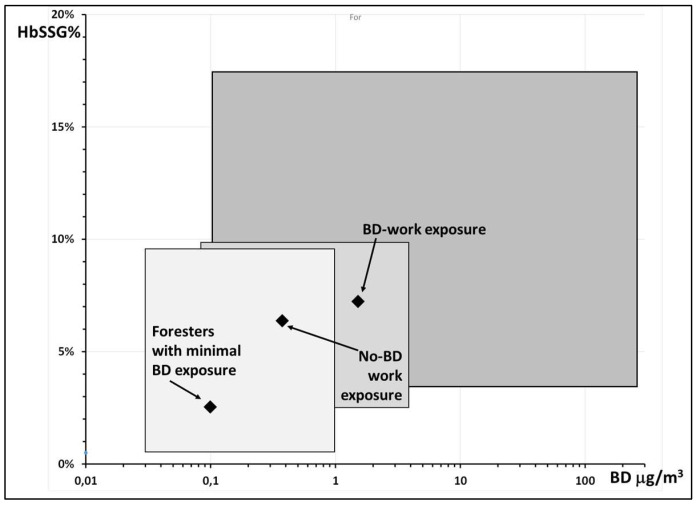
Association of exposure to airborne butadiene (BD) and levels of HbSSG%, reconstructed from the data reported in Table 2 and Figure 1A,D of ref. [27] for the three groups of examined subjects. The solid diamonds identify the median–median position within the boxed intervals; the arrows indicate the three exposure classes. In the three boxes, for BD, the reported interquartile range is used; for HbSSG%, the reported min-max interval is used.

Although the original results are now unavailable, Figure 8 shows a possible relationship of butadiene exposure and HbSSG levels as obtained by compounding the data in box plots A (butadiene exposure) and D (HbSSG% levels) of the original publication. Notwithstanding the limitations of this approach, higher levels of exposure to butadiene are associated with higher levels of HbSSG.

In addition, as reported, measured activity of both GST iso-forms consistently decreases with increasing exposure to butadiene [27]. Overall, this observation is also consistent with a toxic mechanism of butadiene that involves enzyme inactivation.

## 3. Discussion

The re-analysis of published data in the literature, obtained in mutually independent studies that had different aims, can be useful in highlighting some important aspects of the biological role of glutathionyl-hemoglobin in coping with, and recovering from, the oxidative stress of RBCs in subjects exposed to acute or chronic challenges from endogenous and exogenous reactive chemicals or physical stressors.

### 3.1. Processes Leading to Hemoglobin Glutathionylation

Glutathionylation of hemoglobin occurs from the reaction of reactive, electrophilic and radical sulfur species with nucleophilic thiolate groups supplied by the abundant intra-erythrocyte thiol pool, to generate a disulfide-bridged species [28]. The thiol pool is mainly constituted of the hemoglobin cysteines (the tetrameric protein being at a concentration of approximately 5.5 mM in the cytoplasm of the RBCs) and of glutathione (genetically controlled levels between 0.5 and 3.5 mM [21]). The electrophilic sulfur species are, in particular, under the form of sulfinic acid (R-S-OH) formed either from glutathione or from the β-C93 thiolate group of hemoglobin. Hemoglobin can react even in vitro with hydrogen peroxide to generate Hb-S-OH and, by reaction with glutathione, glutathionyl-hemoglobin [12]. Several oxidants, both oxygen-centered (superoxide anion radical, hydroxyl radical, hydrogen peroxide) and nitrogen-centered (nitric oxide radical, nitrite anion [11]) can react with the accessible thiolate groups of hemoglobin and with glutathione in the RBC cytoplasm to yield sulfur-oxidized species [29,30,31].

Even more interestingly, some processes that lead to the formation of glutathionyl-hemoglobin are not of “generic” oxidative stress due to the indiscriminate exposure to xenobiotic substances, but they show a strong discriminative effect, even within chemical classes. One such example is the systematic in vitro study of aromatic quinones present in tobacco smoke that are able to modulate, essentially to impair, the oxygen transport and release properties of hemoglobin in RBCs. Of four studied analogues, only phenanthrene-quinone caused the formation of glutathionyl-hemoglobin, while the others only formed the conjugation thioether with glutathione and the adduct with the β-chain of hemoglobin, but no measurable glutathionyl-hemoglobin [32].

Several parallel oxidative processes occur continuously in the RBCs, where soluble oxidizing species are inactivated by enzymic cascades, such as the inactivation of superoxide by tandem conversion to hydrogen peroxide by superoxide dismutase and the degradation of generated hydrogen peroxide by catalase. Oxidant species that escape degradation react with biological structures, such as with unsaturated membrane lipids, to produce lipid hydroperoxides, which can be in turn transformed into less destructive species by soluble antioxidants, especially by glutathione, the oxidized form of which, sulfinic acid, can next react with other thiols (glutathione thiol and hemoglobin) to generate the respective disulfides (glutathione disulfide and glutathionyl-hemoglobin, respectively) or be directly reduced by the glutathione-reducing enzymatic systems [29].

**Figure 9:**
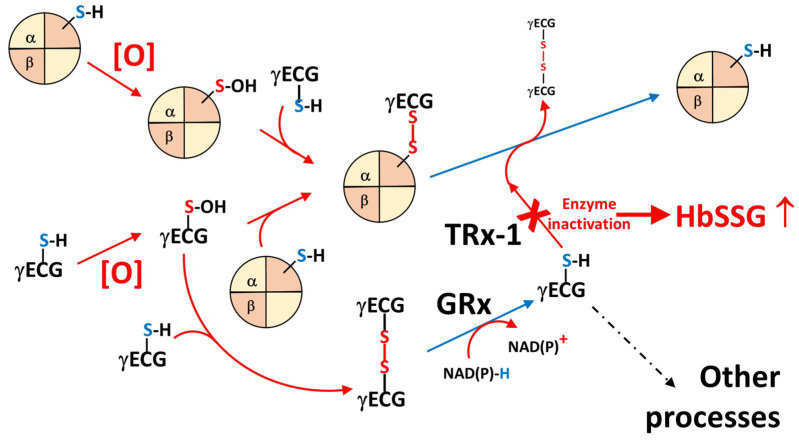
Main general oxidative stress processes that generate and reduce glutathione disulfide and glutathionyl-hemoglobin in red blood cells (γECG = glutathione).

In turn, glutathionyl-hemoglobin, which is either formed directly from hemoglobin-sulfinic acid, from glutathione-sulfinic acid or by disulfide-exchange with glutathione disulfide, is reduced back to the thiol-free form by specific enzymic systems, especially by thioredoxin-1, which needs glutathione as its further reductant and, downstream, NAD(P)H as the terminal electron donor [33,34]. Figure 9 illustrates part of this complex network of processes.

### 3.2. The Antioxidant Proteome and Other GS-Related Enzymes in the Mature Red Blood Cell

The recent studies of quantitative assessment of the proteome of human RBCs [35] and of the intracellular protein assembly [36] are vital to forward hypotheses on the possible recovery mechanism of hemoglobin from its glutathionylated form and on the reasons for its impairment.

**Figure 10:**
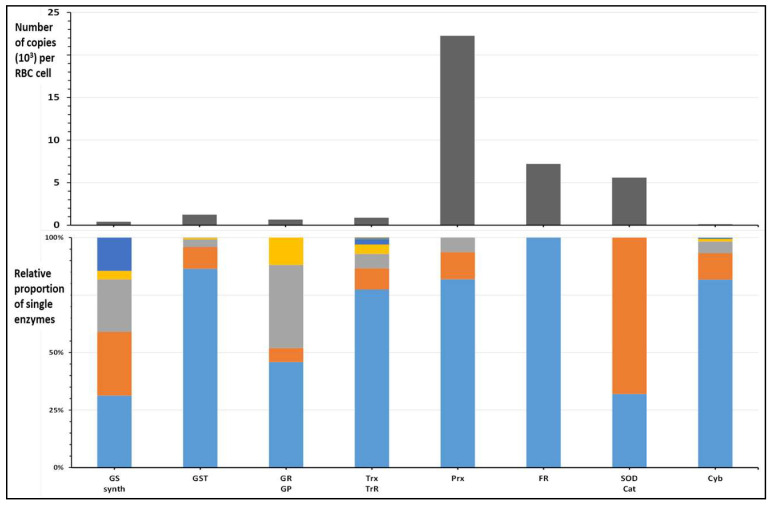
Relative amounts of some enzyme groups (GS: glutathione synthesis; GST: glutathione S-transferases; GR, GP: glutathione reductase and peroxidase; Trx, TrR: thioredoxin and thioredoxin reductase; Prx: peroxiredoxin; FR: flavin reductase; SOD, Cat: superoxide reductase, catalase; Cyb: cytochrome b and cytochrome b reductase) involved in the synthesis, use and redox homeostasis of glutathione and hemoglobin in the RBC proteome, expressed as sum of the copy numbers reported in [35] (**above**) and distribution of the individual proteins within the group (**below**). Data are reported in Table 2.

In the results table published by Bryk and Wisniewski (Supplemental Table 3 of [35]), more than 2500 proteins are listed, expressed as the calculated copy number in whole RBCs (for reference, hemoglobin β-chain is estimated at 180 million copies/RBC cell). The 37 enzymes (out of more than 2000 proteins quantified in the database) involved in the coping mechanisms of oxidative stress, and mostly employing glutathione as the soluble mediator of the response, are classified in eight groups, as summarized in Table 2. The relative levels, expressed as copy number/RBC cell, of a few enzymes involved in RBC redox homeostasis and in the production, use and redox recycling of glutathione and hemoglobin were extracted from the database and are reported in the histograms of Figure 10 for better comparison.

Mature, a-nucleated human erythrocytes are considered to not have any residual protein synthesis activity, and thus, any permanent inactivation of enzymes depletes the red blood cells of their capacity to tackle further environmental challenges, until their vital capacity is gradually exhausted and the now swollen cells are naturally cleared from circulation and replaced with newly matured ones that possess a likely intact suite of enzymes. However, while very recent data support the existence of residual protein biosynthesis capacity in the mature RBCs, with a lower (approx. 10%) production rate than in reticulocytes, this process is limited to the main globins, and there is no evidence of mRNAs for other proteins, including enzymes [37].

### 3.3. Processes Leading to Hemoglobin De-Glutathionylation

**Figure 11:**
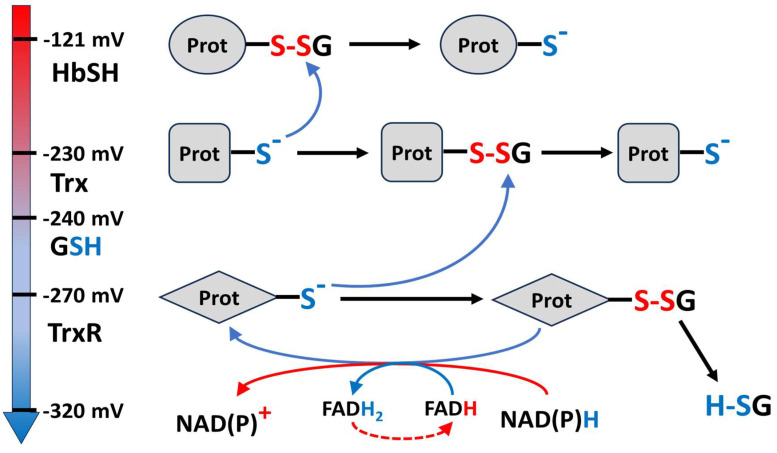
A qualitative scheme of the “mono-thiol” reduction pathway of glutathionyl-hemoglobin by the trans-glutathiolation enzyme cascade and final glutathione reduction by the NAD(P)H terminal hydride donor. The values of the normal redox potential for thioredoxin (Trx) and thioredoxin reductase (TrxR) are those reported in [42], and that of glutathione is shown for reference.

The removal of the glutathione unit from glutathionylated hemoglobin employs the protein disulfide/sulfide housekeeping machinery of the RBC. In the general reduction cascade, a glutathionylated protein transfers—by a nucleophilic trans-thiolation reaction—the disulfide-bound glutathione unit to the thiol group of a catalytic protein with a more negative redox potential. In turn, the last protein of the series, that with the more negative redox potential, reduces the disulfide bond by a formal hydride transfer from the NAD(P)H/NAD^+^//FADH_2_/FADH system, which is the terminal, most negative intracellular redox pair, as illustrated in Figure 11 for the as yet accepted redox sequence of hemoglobin de-glutathionylation [38,39].

One caveat in this discussion is that the values of the redox potentials of the different proteins that are reported in the literature are relative to analogs that may differ from those specific to the human erythrocyte. This is an important aspect to be considered, since the span of normal redox potentials of the same nominal protein can be wide among different iso-forms, and its value depends, even strongly, on limited differences in the amino acid sequence [40,41].

The activity of several proteins that include cysteine thiol groups as part of their active site can be regulated by glutathionylation, a process which temporarily and reversibly inactivates their action. In addition, glutathionylation of other “free” cysteines can modify the conformation and influence enzymatic activities. This complex behavior extends to other biochemical processes that depend on the redox state of the cells, such as “redox sensing” and “redox regulating” the cell’s response to the external environment [38].

The main enzymatic system that deals with the reduction of glutathionyl-hemoglobin is constituted of two enzymes, thioredoxin and thioredoxin-reductase [33,34], the levels of which, as a fraction of hemoglobin, are less than 1% or are less than micromolar intracellular concentrations.

The most abundant thioredoxin iso-enzyme (Trx, UniProt P10599) is present at 680 thousand copies, and the tandem enzyme, thioredoxin reductase (TRxR, UniProt Q16881), is present at less than 20 thousand copies [37]. It is thus conceivable that electrophiles or irreversible oxidants, even only in minute amounts, can, in principle, inactivate a substantial fraction of the available enzyme content, thus seriously impairing the recycling of disulfide-modified proteins, in particular of glutathionylated hemoglobin.

Another, even more abundant group of iso-enzymes is that of Peroxyredoxins, the main isoform of which, Prx-2 (Thioredoxin peroxidase, UniProt P32119), is present at a much higher level of 18 million copies (the enzyme is present as a decamer [43,44], all units being functional units), with the scope of directly scavenging hydrogen peroxide by forming dimeric disulfides (both intermolecular, “typical” and intra-molecular, “atypical” [43]) that are then reduced by thioredoxins [44]. Another class of Prx isoenzymes only carries a single cysteine residue and can operate with a different mechanism, which entails the formation of a thiyl radical (Prot-SH → Prot-S^•^) when interacting with and “quenching” radical species of oxygen and nitrogen, then being reduced through a mechanism that entails the essential intermediacy of ascorbic acid, through its ascorbate radical anion species [43,44,45].

Within the RBC’s proteome, although Trx-1 is a relatively low-expressed enzyme in the mature RBCs, its activity as the main trans-thiolating and reducing agent of protein mixed disulfides, especially with glutathione, is in a nodal position in the network of antioxidant enzymes [46,47,48]. In fact, it also operates as the trans-thiolating agent for the most abundant class of erythrocyte redox proteins, the Peroxyredoxin isoenzyme family, of which Prx-2 is the most abundant.

It is conceivable that, considering the comparatively lower level (at least one order of magnitude lower, as protein copies, estimated in Table 2) of Trx, which is necessary to recycle oxidized Prx-2, HbSSG and oxidized Prx-2, would compete for reduction by Trx. Likely, any lower availability of Trx and TrxR, either due to inhibition or inactivation of the enzymes by enhanced levels of chemical stressors, or due to competition for reduction of high levels of oxidized Prx, would delay the reduction of HbSSG, thus increasing its measured level in oxidatively stressed RBCs.

The efficiency of de-glutathionylation of mixed-disulfide proteins by the enzyme cascade depends both on enzyme thiol-transfer characteristics, expressed as the respective “normal potential” [41], and on their effective intra-erythrocyte concentration where the reaction occurs, in the free cytoplasm or where enzymes are tethered to the cytoskeleton [30].

### 3.4. Modulation of Hemoglobin de-Glutathionylation and HbSSG Levels

When the necessary enzyme systems are impaired, an accumulation of glutathionyl-hemoglobin occurs. Several environmental, lifestyle, physio-pathologic and occupational stressors produce reactive endogenous or exogenous reactive substances that can impair enzyme activity by covalent inactivation of their key functional features. One main target of their electrophilic reactivity is the same functional group, the accessible β-93 cysteine thiolate, which gives rise to thioether protein–electrophile adducts [49], as well as to mixed disulfide. Smoking is one of the main lifestyles that contributes to premature aging of RBCs, in which hemoglobin shows the signs of irreversible damage due to diverse reactive substances [18,19,20,50,51,52,53,54].

As exemplified by the presented study cohort, both main erythrocyte antioxidant systems are impaired and contribute to displacing the redox potential towards less negative, pro-oxidant values. Since antioxidant systems based on thiol-disulfide redox equilibria are based on cysteine-containing enzymes, several reactive chemicals contained in tobacco smoke may covalently modify and inactivate the reactive centers of the enzymes [52,53].

**Figure 12:**
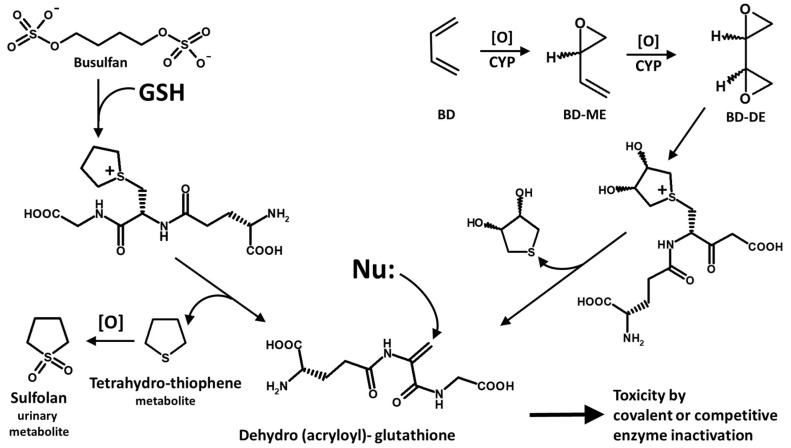
Scheme of the structures and metabolic relationship of busulfan (**left**) and butadiene (**right**) with the acrylate electrophilic derivative of glutathione.

To understand the role of enzyme inactivation as a possible mechanism responsible for the increase of the levels of glutathionyl-hemoglobin, the two cases represented by the patient treated with busulfan (Figure 7) and the petrochemical plant workers exposed to 1,3-butadiene (Figure 8) represent useful examples of the specific action of the two substances. A peculiar behavior of busulfan (left) and butadiene (right) is their biotransformation by glutathione, which is outlined in Figure 12.

The spontaneous degradation of the cyclic addition product of busulfan with glutathione (processes on the left) occurs by beta-elimination and generates tetrahydrothiophene (which is oxidized and eliminated as sulfolane) and a glutathione-derived tripeptide that carries a “dehydroalanine” functionality with a strong electrophilic character in place of the cysteine residue (glutathione *Umpolung*). This functional unit closely resembles acrylamide in its strong electrophilic reactivity and interacts with, and covalently binds to, enzymes that have a glutathione-hosting cleft, such as the detoxifying enzyme glutathione S-transferase [26], likely at its active-site Cys residue [54]. In addition to its glutathione conjugating activity that transforms electrophiles into thioethers, the active-site 32Cys residue of human GST-Omega (UniProt P78417) may show a de-glutathionylating activity [55], although its relevance in the frame of the HbSSG levels within the butadiene exposure cohort cannot be further investigated.

Therefore, busulfan and butadiene (through the diepoxide metabolite) essentially convert the nucleophilic antioxidant and electrophile-quenching glutathione into a nucleophile-quenching molecule capable of functionally inactivating enzymes and suppressing the modulation of biological responses that depend on reversible glutathionylation as the regulating motif. It is of further interest to highlight that the thiyl radical of cysteine peptides, such as of glutathione, can evolve into the same species [56].

An analogous process can occur from butadiene, through its diepoxide (processes on the right in Figure 12). Interestingly, there is strong experimental evidence that the biotransformation of olefins into their epoxides can be directly obtained in the RBCs by oxy-hemoglobin, as demonstrated for styrene [57,58,59], directly at the site of butadiene inhalation, rather than, or in addition to, participation of the liver as the bio-transforming organ.

In the analysis of the recovery from transient oxidative stress, only the metrics based on calculation of the redox potentials and phase space analysis of the recovery pathway (Figure 2 and Figure 5) allowed for the identification of different mechanisms for GSSG and HbSSG reduction. The first-derivative elaboration of Figure 5 highlighted a bi-modal mechanism for the latter, with the possible necessity of a “threshold” level of GSH and of sufficient enzyme activity for the reduction of HbSSG to proceed towards a complete recovery [46].

Of course, validation of each hypothesis, such as those forwarded, based on in vivo exposure of human RBCs to busulfan and butadiene, need experimental validation that should derive from redox proteomic studies of RBC enzymes at the different levels of oxidative stress, along the recovery trajectory of experiments such as that with t-butyl hydroperoxide, with other mechanistically specific chemical toxins, and also in conditions of chronic oxidative stress. A useful concept in this frame is the degree of occupation of “free” redox-active cysteines by glutathione [60].

## 4. Materials and Methods

This article uses data mostly obtained in previously published articles, and mainly, further calculations are the object of this work. Table 3 summarizes the considered cohorts, the source of the data and the methods employed for the measurements, in the same order they are discussed in the Section 2.

The full description of the studies is reported in the cited references.

The first study [16] challenged in vitro red blood cells from a healthy human donor with a radical-generating oxidant and measured the levels of glutathione (GSH), glutathione disulfide (GSSG) and glutathionylated hemoglobin (HbSSG) before and during the recovery phase from the oxidative burst, for approximately 180 min.

The second study [18,19] examined blood samples from 30 quitting-refractory strong smokers at enrollment (T0) and after 90 days of administration of preparations of “Encapsulated Fruit and Vegetable Juice Powder Concentrates” as a damage-reducing treatment (T1). Among other biomarkers and clinical parameters, soluble thiols in the reduced and oxidized (disulfide) form and HbSSG [20] were measured in the RBC.

The third study examined samples taken from a patient undergoing myelo-suppression treatment with injectable busulfan, preliminary to Bone Marrow Transplantation, over the whole hospital stay and treatment.

The fourth study examined single samples from a study cohort of workers exposed to butadiene in a chemical plant and matched unexposed control subjects [27].

Measurements of the second and third study derive from the Author’s laboratory files. Results of the second study were published as method fitness-for-purpose demonstration [20]. Data of the third study are unpublished.

Data from the literature studies [16,27] were retrieved for further numeric elaboration as such, when already reported in their numerical form, or by digitization of graphs and figures and further manual elaboration. Reasonable assumptions on relevant anatomo-physiological parameters of the subjects were used, where necessary, to perform the calculations.

Glutathionyl-hemoglobin in studies [20] (the Author’s laboratory) and [27] (from the published literature) was measured by Matrix-Assisted Laser Desorption Mass Spectrometry in a Time-of-Flight instrument, according to the respective, cited published methods that underwent technical improvements over time [8,9,20].

In the RBCs of the subjects examined in the second study, the levels of glutathione (GSH) and glutathione disulfide (GSSG) were measured by HPLC in an independent laboratory. The method employed pre-column derivatization of the thiol form with a chromogen and liquid chromatography. Glutathione disulfide was calculated as the difference between total and thiol (no-prior-reduction) glutathione [52].

In the first study, total red blood cell proteins were obtained by hemolysis and precipitation, separated by gel electrophoresis and revealed with a glutathione-specific antibody, followed by chromogenic visualization [16].

Hematocrit (as %) and total blood hemoglobin concentration were measured with clinical laboratory methods.

Calculation schemes for this work were performed with custom Microsoft Excel spreadsheets [17]. Briefly, calculations involve data conversion from the original HbSSG% peak-ratio measurements to the metrics that are necessary to perform further data elaboration and the calculation of the redox potential [17].

The calculation of the redox potential of each redox pair (HbSSG/HbSH and GSSG/GSH) with the Nernst equation uses the value of E_0′_ (referred to pH 7.2) reported in the cited literature for glutathione (−264 mV [13]) and for hemoglobin (−121 mV [16]).

## 5. Conclusions

This reappraisal of published results contributes to highlighting the complex role of HbSSG as one of the nodes in the thiolome redox circuitry of human RBCs. As can be envisaged, in particular, from the ex vivo, in vitro single-strike oxidant challenge experiment, the results strengthen the hypothesis that, under an acute oxidant insult, the first-line response of the thiolome buffer is the transient oxidation of glutathione and the intervention of the larger pool of hemoglobin as a glutathione-sparing scavenger. Once the RBC restores its redox homeostasis after the transient insult, the release of glutathione, at first from the disulfide, next from HbSSG, restores the pre-challenge physiological equilibrium within the thiolome pool.

The outcome of the first study thus represents a very useful reference for RBC resilience in conditions of reversible oxidative stress, when healthy RBCs with fully functional antioxidant systems are tested within the “elastic” limits of response, which correspond to biological homeostasis. In principle, this behavior may be tested in individuals to preventively assess their resistance to conditions entailing otherwise unavoidable oxidative stress, such as from surgical treatments [13], sport or working activities in extreme environments [63,64].

Using the E_h_’ “reference line” of HbSSG can make the assessment of oxidative stress a simpler task compared to other molecular measurements, such as that of the soluble thiols, other biomarkers and functional tests [65]. Experienced laboratories repeatedly warn that very cautious procedures should start at the stage of blood collection to avoid oxidative artifact generation and unreliable results [66]. In our experience, HbSSG can be measured in samples that have been preserved over a long time under the reasonable conditions of bloodletting, processing and storing that can be achieved even in field studies [63,64].

Interpretation of the results from the observational–interventional group study of heavy tobacco smokers strongly suggests that individuals have different levels of resilience to oxidative stress that are linked both to their genetically determined levels of glutathione and to the extent of impairment to the antioxidant defense mechanisms, especially those based on enzymes. In RBCs, this phenomenon is particularly apparent, due to the lack, in the a-nucleated mature erythrocytes, of most protein repair mechanisms and supplementary intracellular protein synthesis of the antioxidant enzymes. Therefore, RBCs represent a still untapped resource for holistically testing the dynamic resilience of the individual human body to oxidative stress challenges that can occur in a host of different unexpected exposure scenarios [67], lifestyles, chemical exposures, extreme environmental conditions, and scenarios of occupational relevance, such as shift work [68,69].

In addition, biomarker stability, relatively easy measurement and an expanded suite of metrics, such as that based on redox potentials, can encourage the use of glutathionyl-hemoglobin to quantitatively assess subject resilience, response to oxidative stressors and the efficacy of coping measures [70], such as lifestyle, dietary and nutraceutical interventions [71], aimed at facing the effects of pro-oxidant conditions in everyday life.

## Figures and Tables

**Table 1:** Treatment schedule of the Bone Marrow Transplantation patient.

	Day ^1^	Hour	Treatment	HbSSG% ^2^
1	−6	11:00	Admission to transplant unit	3.3
2	−6	15:00	End of first BUS infusion	2.3
3	−3	12:15	Prior to second BUS infusion	3.1
4	−3	15:40	End of second BUS infusion	2.8
5	−2	11:30	Recovery day 1	3.0
6	−1	9:40	Recovery day 2	3.6
7	0	9:30	Heterologous Bone Marrow Transplantation	5.0
8	+3	9:00	Post-transplantation recovery day	5.5
9	+4	9:00	Post-transplantation recovery day	4.1
10	+5	9:00	Post-transplantation recovery day	3.1
11	+6	9:15	Post-transplantation recovery day	4.6
12	+7	9:20	Post-transplantation recovery day	4.5
13	+10	9:20	Post-transplantation recovery day	2.9

^1^ according to clinical practice, the days of patient stay at the hospital are (retrospectively) numbered by assigning day zero to that scheduled for the transplant; ^2^ ES ± 0.6%.

**Table 2:** Main RBC enzymes involved in the synthesis, use and redox homeostasis of glutathione and hemoglobin.

Group	UniProt ^1^	Name	EC	n° Copies ^2^
1	P48506	Glutamate--cysteine ligase catalytic subunit	6.3.2.2	128,678
	Q9BVM4	Gamma-glutamylamine-cyclotransferase	4.3.2.8	113,766
	P48507	Glutamate--cysteine ligase regulatory subunit	6.3.2.2	94,087
	O75223	Gamma-glutamyl-cyclotransferase	4.3.2.9	15,276
2	P78417	Glutathione S-transferase omega-1	2.5.1.18	1,078,139
	P09211	Glutathione S-transferase P	2.5.1.18	117,005
	P30711	Glutathione S-transferase theta-1	2.5.1.18	42,464
	P21266	Glutathione S-transferase Mu 3	2.5.1.18	8056
	P28161	Glutathione S-transferase Mu 2	2.5.1.18	1083
	P09210	Glutathione S-transferase A2	2.5.1.18	273
3	P35754	Glutaredoxin-1		308,769
	O76003	Glutaredoxin-3		40,802
	P07203	Glutathione peroxidase 1	1.11.1.9	243,271
	P00390	Glutathione reductase, mitochondrial	1.8.1.7	79,737
4	P10599	Thioredoxin		678,882
	O43396	Thioredoxin-like protein 1		81,206
	Q9H3N1	Thioredoxin-related transmembrane protein 1	5.3.4.1	54,514
	Q9BRA2	Thioredoxin domain-containing protein 17		35,655
	Q16881	Thioredoxin reductase 1, cytoplasmic	1.8.1.9	20,375
	P30048	Thioredoxin-dependent peroxide reductase, mitochondrial	1.11.1.24	2269
	Q8NBS9	Thioredoxin domain-containing protein 5		2167
	Q9H1E5	Thioredoxin-related transmembrane protein 4		1599
5	P32119	Peroxiredoxin-2	1.11.1.24	18,223,631
	P30041	Peroxiredoxin-6	1.11.1.27	2,638,035
	Q06830	Peroxiredoxin-1	1.11.1.24	1,383,765
	Q13162	Peroxiredoxin-4	1.11.1.24	8352
	P30044-2	Peroxiredoxin-5, mitochondrial	1.11.1.24	5393
6	P30043	Flavin reductase (NADPH)	1.5.1.30	7,201,284
7	P00441	Superoxide dismutase [Cu-Zn]	1.15.1.1	1,791,289
	P04040	Catalase	1.11.1.6	3,799,230
8	P00167-2	Cytochrome b5		95,072
	Q53TN4	Cytochrome b reductase 1	7.2.1.3	13,337
	J3KNF8	Cytochrome b5 type B		5928
	O14569	Cytochrome b561 domain-containing protein 2	7.2.1.3	1349
	P31930	Cytochrome b-c1 complex subunit 1, mitochondrial		511
	P22695	Cytochrome b-c1 complex subunit 2, mitochondrial		105

^1^ first UniProt code assigned in the database [35]; ^2^ sum of RBC copy number (avg.) and White Ghost (WG) copy number (avg.).

**Table 3:** Cohorts used in this data re-analysis, sources of the data and measurement methods employed.

Study ^1^	Cohort ^1^	n_subj	HbSSG (n° Samp; Method)	Glutathione (n° Samp; Method)	Notes
1 [13]	*t*-Bu-HP	1	13; HPLC [16]	12; HPLC [61]	In vitro experiment
2 [15,17]	Smokers	30	60; MALDI-ToF [19]	60; HPLC [62]	Pre- and post-treatment
3	Busulfan	1	7; MALDI-ToF [19]	no	Single subject, unpubl.
4 [20]	butadiene	109	109; MALDI-ToF [8]	no	42 NE + 43 LE + 24 HE ^2^

^1^ follows the order of the Section 2; ^2^ NE: not exposed (“Foresters”); LE: low-exposure (plant office workers); HE: high exposure (petrochemical plant employees).

## Data Availability

Calculation spreadsheets are available from the author upon reasonable request.
